# Diversification of Fungal Specific Class A Glutathione Transferases in Saprotrophic Fungi

**DOI:** 10.1371/journal.pone.0080298

**Published:** 2013-11-20

**Authors:** Yann Mathieu, Pascalita Prosper, Frédérique Favier, Luc Harvengt, Claude Didierjean, Jean-Pierre Jacquot, Mélanie Morel-Rouhier, Eric Gelhaye

**Affiliations:** 1 Université de Lorraine, IAM, UMR 1136, IFR 110 EFABA, Vandoeuvre-les-Nancy, France; 2 INRA, IAM, UMR 1136, Champenoux, France; 3 Université de Lorraine, CRM2, UMR 7036, Vandoeuvre-les-Nancy, France; 4 CNRS, CRM2, UMR 7036, Vandoeuvre-les-Nancy, France; 5 Laboratoire de biotechnologie, Pôle Biotechnologie et Sylviculture Avancée, FCBA, Campus Forêt-Bois de Pierroton, Cestas, France; UC Irvine, United States of America

## Abstract

Glutathione transferases (GSTs) form a superfamily of multifunctional proteins with essential roles in cellular detoxification processes and endogenous metabolism. The distribution of fungal-specific class A GSTs was investigated in saprotrophic fungi revealing a recent diversification within this class. Biochemical characterization of eight GSTFuA isoforms from *Phanerochaete chrysosporium* and *Coprinus cinereus* demonstrated functional diversity in saprotrophic fungi. The three-dimensional structures of three *P. chrysosporium* isoforms feature structural differences explaining the functional diversity of these enzymes. Competition experiments between fluorescent probes, and various molecules, showed that these GSTs function as ligandins with various small aromatic compounds, derived from lignin degradation or not, at a L-site overlapping the glutathione binding pocket. By combining genomic data with structural and biochemical determinations, we propose that this class of GST has evolved in response to environmental constraints induced by wood chemistry.

## Introduction

Glutathione transferases (GSTs; EC 2.5.1.18) constitute a large superfamily of multifunctional enzymes that are involved in phase II of xenobiotic detoxification by catalyzing the conjugation of glutathione (GSH) to a variety of electrophilic compounds [1], [2]. Most of the GSTs are dimeric proteins, each monomer harbouring two domains : a thioredoxin N-terminal domain containing the conserved GSH binding site (G site) and a more variable C-terminal α-helical domain (H site) usually involved in the binding of the GSH acceptor compound [3]. Besides their catalytic activities, some GSTs can also exhibit ligandin properties, involving a so-called L-site. The latter property has been defined as the capacity to bind non-substrate ligands contributing to intracellular sequestration and transport of xenobiotics or hormones [4]. In plants, GSTs could be involved in transport of hydrophobic compounds such as pigments [5]. More recently, the human glutathione transferase omega 1-1 was also shown to possess a L-site binding S-(4-nitrophenacyl) glutathione at the dimer interface and was suggested to be the binding location of uncompetitive inhibitors such as tocopherol [6].

The release of fungal genomes allowed to unravel a specific evolution of cytosolic GSTs in these organisms in correlation with their way of life [2],[7]. Indeed, saprotrophic fungi such as the wood-decayer *P. chrysosporium* or the litter decomposer *C. cinereus* exhibit a high number of GST encoding genes in comparison to symbiotic fungi or biotrophic pathogens. The fungal specific class GSTFuA is particularly concerned by this expansion. For instance, *C. cinereus* possesses a high number of GSTFuA with 14 isoforms representing nearly the half of the total GSTs found in this organism (32 GST encoding sequences), whereas 5 isoforms of GSTFuA, on a total of 25 GSTs, are present in *P. chrysosporium*. In a recent study, we have characterized for the first time a member of this fungal specific class in *P. chrysosporium* (PcGSTFuA1). PcGSTFuA1 displays unique structural and biochemical features, exhibiting overlapping G and L-sites [8].

The aim of this study was to extend the characterization of the GSTFuA class using comparative genomic, biochemical approaches performed on eight GSTFuA proteins (four from *P. chrysosporium* and four from from *C. cinereus*) coupled with structural studies of three isoforms from *P. chrysosporium*.

## Materials and Methods

### Material

Hydroxyethyldisulfide (HED) was from Pierce. 5-chloromethylfluorescein diacetate (CMFDA) was from Invitrogen. S-(phenylacetophenone)-glutathione (PAP-SG) and 2-methyl-S-glutathionyl-naphtoquinone (menadione-SG) were synthesized as previously described [9]. All other reagents were from Sigma-Aldrich.

### Cloning of fungal GSTFUAs

The open reading frame sequences were amplified from *P. chrysosporium* and *C. cinereus* cDNA libraries using forward and reverse primers (Table 1), and cloned into the NcoI and BamHI restriction sites (underlined in the primers) of pET-3d (Novagen). The amplified sequences encoded proteins in which an alanine has been inserted after the initiator methionine to improve protein production.

**Table 1:** Primers used in this study.

	Sequence
PcGSTFUA1 for	5' CCCCCCATGGCTCAGCCCATCGTGTTC 3'
PcGSTFUA1 rev	5' CCCCGGATCCCTATACATCAACCTGCTC 3'
PcGSTFUA2 for	5' CCCCCATGGCTTCCCAGCCCATTGTCTTC 3'
PcGSTFUA2 rev	5' CCCCGGATCCTTAGTCATCTGCCCGCTC 3'
PcGSTFUA3 for	5' CCCCCATGGCTTCCCTCGAGCCCATCATC 3'
PcGSTFUA3 rev	5' CCCCGGATCCCTAGACGTCTACGAACTC 3'
PcGSTFUA4 for	5' CCCCCATGGCCGATGTTATCACCCTGTACG 3'
PcGSTFUA4 rev	5' CCCCGGATCCCTACAGGTCTACATGCGC 3'
CcGSTFUA2461 for	5' CCCCCCCATGGCAATAACCTTCTACGACCTA 3'
CcGSTFUA2461 rev	5' CCCCCGGATCCTACAATTTACTTCCCGT 3'
CcGSTFUA6800 for	5' CCCCCCCATGGCAATTATACTATACGACCTC 3'
CcGSTFUA6800 rev	5' CCCCCGGATCCTTACACAACAGTAGTATA 3'
CcGSTFUA6801 for	5' CCCCCCCATGGCAATCACCTTCTACGATATC 3'
CcGSTFUA6801 rev	5' CCCCCGGATCCTTATGCGACAGTGTGATA 3'
CcGSTFUA6820 for	5' CCCCCCCATGGCAATCACCCTCTACGACTCC 3'
CcGSTFUA6820 rev	5' CCCCCGGATCCTAATGAACAGCCTGATA 3'

The *Nco*I and *Bam*HI cloning restriction sites are underlined in the primers.

### Expression and purification of the recombinant proteins

For protein production, the *Escherichia coli* BL21(DE3) strain, containing the pSBET plasmid, was co-transformed with the recombinant plasmids [10]. Cultures were progressively amplified up to 2 L in LB medium supplemented with ampicillin and kanamycin at 37°C. Protein expression was induced in the exponential phase by adding 100 µM isopropyl β-D-thiogalactopyranoside for 4 h at 37°C. The cultures were then centrifuged for 15 min at 4400×*g*. The pellets were resuspended in 30 mL of TE NaCl (30 mM Tris-HCl, pH 8.0, 1 mM EDTA, 200 mM NaCl) buffer. Cell lysis was performed on ice by sonication (3×1 min with intervals of 1 min), and the soluble and insoluble fractions were separated by centrifugation for 30 min at 27,000×*g* at 4°C. The soluble part was then fractionated with ammonium sulphate in two steps, and the protein fraction precipitating between 40 and 80% of the saturation contained the recombinant protein, as estimated by 15% SDS-PAGE. The protein was purified by size exclusion chromatography after loading on an ACA44 (5×75 cm) column equilibrated in TE NaCl buffer. The fractions containing the protein were pooled, dialyzed by ultrafiltration to remove NaCl, and loaded onto a DEAE-cellulose column (Sigma) in TE (30 mM Tris-HCl, pH 8.0, 1 mM EDTA) buffer. The proteins were eluted using a 0–0.4 M NaCl gradient. Finally, the fractions of interest were pooled, dialyzed, and concentrated by ultrafiltration under nitrogen pressure (YM10 membrane; Amicon). Purity was checked by SDS-PAGE. Protein concentrations were determined spectrophotometrically using a molar extinction coefficient at 280 nm of 68870 M^−1^.cm^−1^ for PcGSTFuA1, 67380 M^−1^.cm^−1^ for PcGSTFuA2, 58900 M^−1^.cm^−1^ for PcGSTFuA3, 75860 M^−1^.cm^−1^ for PcGSTFuA4, 68410 M^−1^.cm^−1^ for CcGSTFuA2461, 67380 M^−1^.cm^−1^ for CcGSTFuA6800, 69900 M^−1^.cm^−1^ for CcGSTFuA6801 and 66350 M^−1^.cm^−1^ for CcGSTFuA 6820.

### Sequence analysis

All sequences were retrieved from the Joint Genome Institute (JGI) database (http://genome.jgi-psf.org/programs/fungi/index.jsf) from the fungal genomic database MycoCosm. The sequences have been obtained with Blastp (BLOSUM matrix applying default parameters) using all *P. chrysosporium* GSTFuA sequences as template. Fungi showed in the phylogenetic analysis have been chosen according to their saprotrophic properties. The sequence of PcGSTFuA4 has been modified compared to the one available on JGI and the new sequence has been deposited to Genbank under the KC192375 identity number. Sequence alignments have been done using ClustalW and phylogenetic analysis were conducted using Muscle and MEGA5 (Neighbour-joining) programs [11]. The pairwise deletion option was active to handle alignment gaps and the Poisson correction model was used for distance computation. Bootstrap tests were conducted using 1000 replicates.

### Activity measurements

The activities of purified proteins in thiol transferase activity with hydroxyethyldisulfide assay or for reduction of dehydroascorbate (DHA) were performed as described by Couturier and coworkers [12]. The GSH transferase activity was assessed with phenethyl isothiocyanate (phenethyl-ITC) prepared in 2% (v/v) acetonitrile, 1-chloro-2,4-dinitrobenzene (CDNB) and 4-nitrophenyl butyrate (PNP-butyrate) prepared in DMSO. For these three substrates the reactions were monitored respectively at 274 nm, 340 nm and 412 nm following the increase in absorbance arising from the formation of the S-glutathionylated adduct. The reactions with CDNB and PNP-butyrate were performed in 100 mM phosphate buffer, pH 7.5, in presence of GSH (5 mM) while the reaction with phenethyl-ITC was performed at pH 6.5 with an identical GSH concentration. Peroxidase activities were monitored as follows: 1 mM peroxide (hydrogen peroxide, terbutyl hydroperoxide or cumene hydroperoxide) in 30 mM Tris-HCl, pH 8.0, was incubated in presence of 2 mM GSH, 200 µM NADPH, 0.5 IU glutathione reductase. The activity was followed by monitoring the decrease in absorbance arising from NADPH oxidation in this coupled enzyme assay system showing the formation of oxidized glutathione (GSSG). All the reactions were started by addition of the purified enzyme (500 nM) and monitored with a Cary 50 UV-Visible spectrophotometer (VARIAN). To determine the catalytic properties of the enzyme, steady state assays were performed using variable substrate concentrations (from 10 µM to 10 mM) and the catalytic parameters were calculated using the GraphPad® software.

### 8-anilino-1-naphtalene sulfonic acid (ANS) binding and ligand screening

All binding and competition experiments were performed in TE buffer pH 8.0. ANS binding onto purified proteins was investigated by monitoring fluorescence upon addition of various ANS concentrations, depending on the enzyme, to 3 µM protein using a fluorescence spectrophotometer Cary Eclipse (VARIAN). The excitation wavelength was set at 385 nm and the emission spectra recorded from 400 to 700 nm. Additional tryptophan-based fluorescence experiments were performed in presence or absence of ANS. After excitation at 290 nm, emission spectra were recorded from 305 to 550 nm. ANS binding at the H-site was investigated in the same conditions with 50 µM CMFDA.

The correlation between ANS concentration and fluorescence yield was obtained by incubating, 100 µM of enzyme with 1 µM ANS, giving the maximal value for 1 µM ANS bound to the protein. Fluorescence from samples containing ANS alone was subtracted as a background. Proteins were then incubated at a concentration of 3 µM with increasing ANS concentrations ranging from 0 to 1 mM. To determine the dissociation constant of ANS the following equation (Equation 1) was applied y  =  Bmax * [Free ANS]/(Kd + [Free ANS]) where y is the specific binding, i.e. the concentration of ANS bound per protein and Bmax the maximum number of specific binding sites.

Competition experiments were performed in a total volume of 250 µL containing 3 µM enzyme, variable ANS concentration depending on the enzyme, and various putative ligands (from 1 µM to 10 mM). Fluorescence emission from protein-free samples containing ANS and potential ligand were subtracted as background. A decrease in fluorescence, indicating competition between ligand and ANS, was measured after 10 min of incubation with excitation at 385 nm and emission at 475 nm on a VICTOR™ *X*5 plate reader (PerkinElmer). IC50 values were obtained by fitting data to the equation (Equation 2) Y = Bottom+(Top−Bottom)/(1+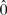
(Log[Ligand]−Log IC50)) where Y is the fluorescence signal observed after background subtraction, Top is the fluorescence signal of ANS without ligand and Bottom the fluorescence signal of ANS at the highest ligand concentration.

### Esterase activity: fluorescence based assays

Esterase activity was measured in microplates using 5-chloromethylfluorescein diacetate (CMFDA) or 4-Methylumbelliferyl acetate (MUA) as substrates, the cleavage of which releases the fluorescent compounds fluorescein and 4-methylumbelliferone respectively. Experiments were performed on a VICTOR™ *X*5 plate reader (PerkinElmer) in 50 mM phosphate buffer pH 8.0 for CMFDA and 30 mM Tris-HCl buffer pH 8 for MUA. Both assays were carried out in a total volume of 200 µL. The reactions were started by addition of the purified enzyme and fluorescence was measured every minute for 1 hour with excitation at 485 nm and emission at 535 nm for CMFDA. For MUA, excitation and emission were at 355 nm and 460 nm respectively. Catalytic parameters were calculated using the GraphPad® software from steady state experiments performed without or with 5 mM GSH using substrates ranging from 0.25 to 50 µM for CMFDA and 10 to 800 µM for MUA.

### Inhibition Kinetics

Competition assays between CcGSTFuAs and potential ligands were performed by measuring esterase activity on MUA without glutathione. Activities were monitored in a total volume of 200 µL containing 0 to 500 µM MUA and 0 to 10 mM of ligand. The reactions were started by the addition of purified enzyme (2 µM), and the catalytic parameters were calculated using the GraphPad® software. For noncompetitive inhibition, K_i_ values were calculated resolving the non-linear noncompetitive inhibition equation: V_maxInh_ = V_max_/(1+I/K_i_); Y = V_maxInh_×X/(K_m_+X). V_max_ and K_m_ represent the maximum enzyme velocity and the Michaelis-Menten constant without inhibitor, whereas V_maxInh_ represents the maximum enzyme velocity for one concentration of inhibitor, and K_i_ is the inhibition constant. For competitive inhibition, the K_i_ value was calculated resolving the non-linear competitive inhibition equation: K_m(obs)_ = K_m_×(1+[I]/K_i_); Y = V_max_×X/(K_m(obs)_+X). V_max_ and K_m_ represent the maximum enzyme velocity and the Michaelis-Menten constant without inhibitor, whereas K_m(obs)_ represents the Michaelis-Menten constant in the presence of inhibitor, and K_i_ is the inhibition constant.

### Crystallization, Data Collection, Structure Determination and Refinement

Crystallization experiments of PcGSTFuA2 and PcGSTFuA3 were performed by the microbatch under oil (paraffin) method at 4°C and 20°C, respectively. Drops were prepared by mixing equal volumes (2 µl) of protein and precipitating agent solutions. TE buffered solutions of PcGSTFuA2 and PcGSTFuA3 had protein concentrations of 20 mg.mL^−1^ and 28.3 mg.mL^−1^, respectively. Suitable crystals for x-ray diffraction were obtained without optimization using Hampton crystallization screen 1. PcGSTFuA2 crystallized in condition 9 (0.2 M ammonium acetate, 0,1 M sodium citrate tribasic dehydrate, pH 5,6 and 30% w/v polyethylene glycol 4,000) and PCGSTFuA3 crystals were obtained in condition 34 (0.1 M sodium acetate trihydrate, pH 4.6, 2.0 M sodium formate solution). X-ray diffraction experiments were performed at 100 K with crystals soaked briefly in crystallization solution supplemented with 20% (v/v) glycerol prior to flash cooling.

X-ray data sets were collected on beamline PROXIMA-1 at the French national Synchrotron facility SOLEIL (Gif-sur-Yvette, France). The data were indexed and processed using XDS [13], scaled and merged with SCALA from the CCP4 program package [14], [15]. Both structures were solved by molecular replacement with MOLREP [16] using the coordinates of PcGSTFUA1 in complex with GSH (Protein Data Bank code 4G19). The models were refined using PHENIX [17] interspersed with manual inspection and corrections using COOT [18]. The validation of both crystal structures was performed with MOLPROBITY [19]. All figures were prepared with PyMOL and crystallographic statistics are available in Table 2. Atomic coordinates and structure factors for PcGSTFuA2 and PcGSTFuA3 have been deposited in the Protein Data Bank (PDBID: 4MLV and 4MLW, respectively).

**Table 2:** Statistics of X-ray diffraction data collection and model refinement.

	PcGSTFuA2	PcGSTFuA3
***Data collection***		
Beam line	PX1, SOLEIL	PX1, SOLEIL
Space group	*C*222_1_	*P*2_1_2_1_2_1_
Number of dimers in the ASU a	3	0.5
Cell dimensions a, b, c (Å)	100.41 202.47 192.76	87.00 87.00 225.70
Resolution (Å)	48.96−3.20 (3.37−3.20) b	45.16−2.10 (2.21−2.10)
Rmerge	0.12 (0.58)	0.064 (0.49)
Mean I/σ (I)	20.7 (4.9)	25.1 (4.0)
Completeness (%)	100.0 (100.0)	99.6 (97.4)
n observations	433,165 (54,032)	441,971 (42,972)
Redundancy	13.2 (11.4)	14.5 (10.2)
Wilson B factor (Å^2^)	78.2	35.9
***Phasing method*** c	**MR**	**MR**
***Refinement***		
Resolution (Å)	48.96−3.20 (3.30−3.20)	45.16−2.10 (2.17−2.10)
n reflections	32,677 (2,679)	30,346 (2,576)
Cutoff	*F*>1.34σ(*F*)	*F*>1.35σ(*F*)
Rall (%) d	19.2	18.8
Rfree (%) d	25.0 (29.6)	21.6 (31.1)
Average B-factor (Å^2^)		
Protein atoms	72.6	38.2
Ligand atoms	70.9	56.6
Solvent atoms	- e	42.2
Ramachandran statistics (%)		
Residues in preferred regions	96.3	98.0
Residues in allowed regions	3.7	1.6
Outlier residues	0.0	0.4
R.m.s.^f^ deviations		
Bond length (Å)	0.017	0.007
Bond angle (°)	1.536	1.085

a ASU : Asymmetric unit.

b Values in parentheses are for highest resolution shell.

c MR : Molecular Replacement.

d *R*all was determined from all the reflections (working set + test set), whereas *R*free corresponds to a subset of reflections (test set).

e No water molecule was added in the PsGTFuA2 3.2 Å model.

f R.m.s. : Root mean square.

## Results

### Genomic analysis of GSTFUA coding sequences

**Figure 1:**
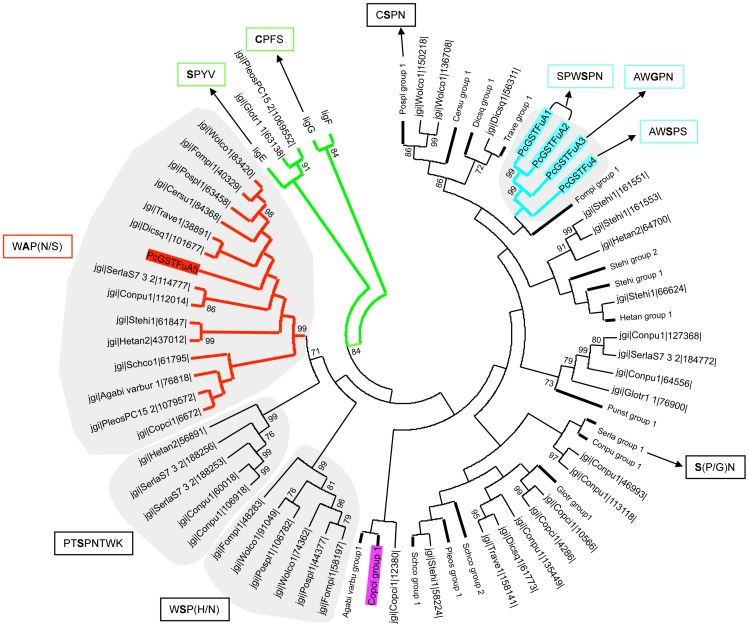
Phylogenetic distribution of GSTFuA sequences in saprotrophic fungi and their bacterial Lig homologues from *Sphingobium* sp SYK-6. Only bootstrap values over 70 are reported and indicated at nodes. Bold bars are compression of sequences from the same organism and boxes indicates putative catalytic residues in bold. PcGSTFuAs and CcGSTFuAs are highlighted in blue and purple respectively while the PcGSTFuA5 group of putative mitochondrial GSTFuAs is highlighted in red. LigEFG are highlighted in green. *Agaricus bisporus* (Agabi varbu) group1 contains: Prot ID 79084, 116016, 74698, 114110, 116585, 108910, 115923, 115917, 122742, 78843, 60492, 115916, 45066, 78836. *Coprinopsis cinerea* (Copci) group1 contains: Prot ID 1283, 2461, 6800, 6801, 4672, 6796, 6820, 6821. *Schizophyllum commune* (Schco) group 1 contains: Prot ID 111982, 59314, 85860. Schco group 2 contains: Prot ID 57691, 114676, 12387, 236992, 81614. *Pleurotus ostreatus* (Pleos) group 1 contains: Prot ID 1074017, 1074021, 1061513, 1103298. *Gloeophyllum trabeum* (Glotr) group 1 contains: Prot ID 109364, 74336, 61485, 76864, 76907. *Coniophora puteana* (Conpu) group 1 contains: Prot ID 98863, 169024, 159836, 113027, 132784. *Serpula lacrymans* (Serla) group 1 contains: Prot ID 94516, 162189, 185784, 112566, 186005, 107446, 780076, 106153, 111690, 185150, 185168, 115219, 188903. *Punctularia strigosozonata* (Punst) group 1 contains: Prot ID 97823, 114205, 50105, 120997, 34993. *Heterobasidion annosum* (Hetan) group 1 contains: 104697, 64692, 426787. *Stereum hirsutum* (Stehi) group 1 contains: Prot ID 161804, 130786, 97768. (Stehi) group 2 contains: Prot ID 125105, 103989, 161719, 171888, 142045. *Fomitopsis pinicola* (Fompi) group 1 contains: Prot ID 127980, 160680, 123507, 80060, 60354, 92199, 92198. *Trametes versicolor* (Trave) group 1 contains: Prot ID 47690, 24779, 64328, 148196. *Dichomitus squalens* (Dicsq) group 1 contains: prot ID 138582, 82843, 102613. *Ceriporiopsis subvermispora* (Cersu) group 1 contains: prot ID 163524, 73205, 83044. *Postia placenta* (Pospl) group 1 contains: Prot ID 87703, 50241, 88670, 50291, 43801, 95829, 91470, 108840.

**Figure 2:**
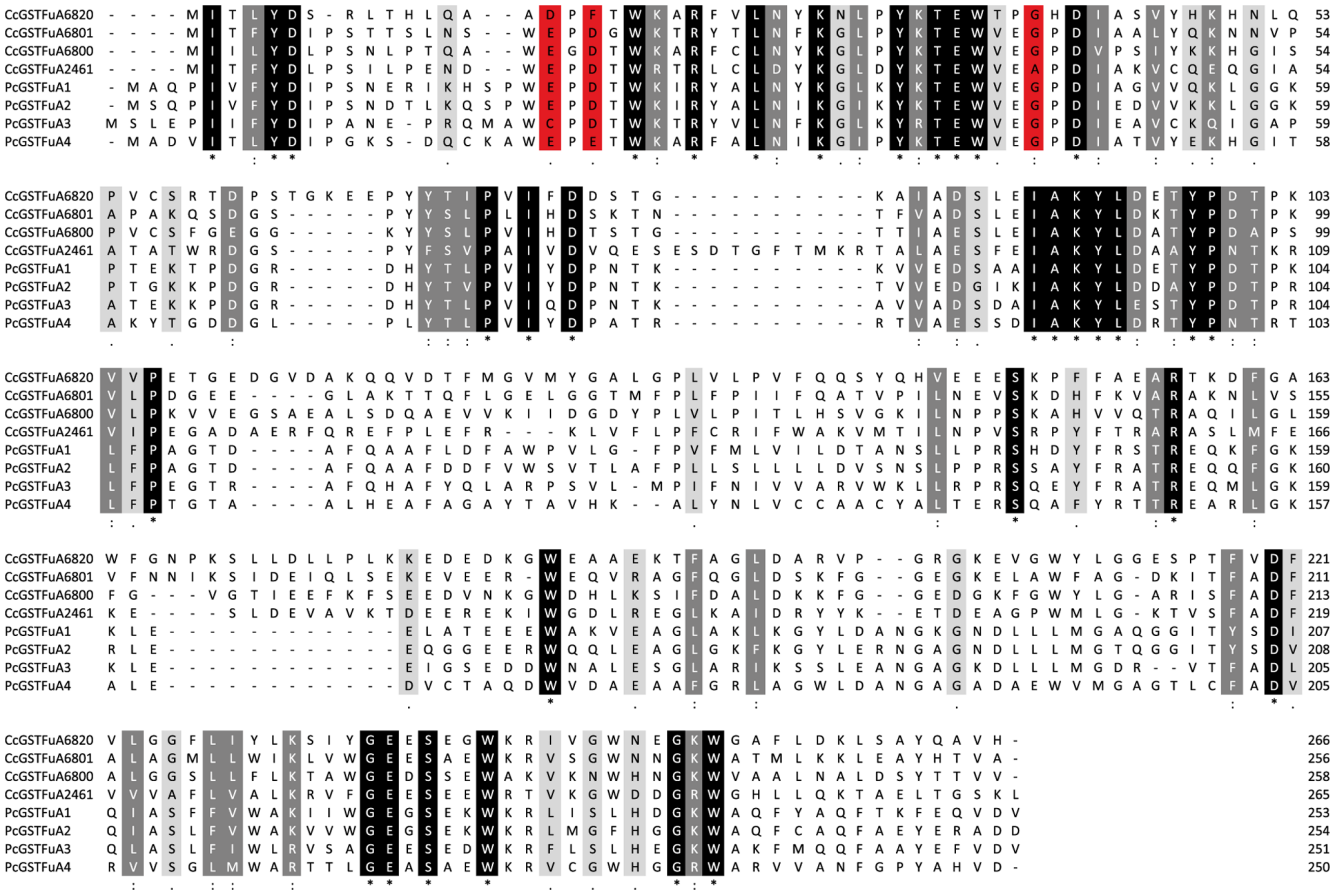
Sequence alignment of GSTFuAs investigated in this study. Alignment was performed with ClustalW, and potential catalytic residues are highlighted in red.

The fungal genomic portal access Mycocosm on JGI is currently providing an increasing dataset of fungal genome sequences. GSTFuA sequences have been collected from the JGI database in all available genomes of Chytridiomycota, Mucoromycotina, Saccharomycotina, Pezizomycotina, Pucciniomycotina, Ustilagomycotina and Agaricomycotina. The number of detected GSTFuA sequences is highly variable between and inside fungal phyla and is not related to the number of predicted gene models in the genome (Table S1). Among Ascomycota, no GSTFuA-related sequence was identified in Saccharomycotina genomes, while 0 to 6 sequences were found in Pezizomycotina genomes. Similarly, among Basidiomycota, GSTFuA sequences were only detected in Agaricomycotina genomes (from 0 to 17 sequences). The distribution of this GST class is thus not correlated to fungal taxonomy but rather to the fungal way of life since, for instance, wood interacting fungi exhibit 2 to 17 GSTFuA sequences according to the species, whereas pathogens, such as *Puccinia graminis*, do not display any GSTFuA sequences. The phylogenetic analysis presented in Figure 1 focuses on Agaricomycotina GSTFuAs, including also some bacterial etherases (LigE and LigF from *Sphingobium* sp. SYK-6) since these proteins involved in lignin breakdown display homology with GSTFuAs. Globally, the sequences cluster according to the organism. However, a specific subclass could be identified which includes PcGSTFuA5 and its homologues. One isoform belonging to this subclass has been identified in each studied genome, except in *Gloeophyllum trabeum* and *Punctularia strigosozonata*. The putative proteins of this subclass are predicted to be located in mitochondria, suggesting that they could have a conserved function in this organelle. Figure 1 highlights the amino acids surrounding the putative catalytic residue. Most of the sequences exhibit a serine, which is known to be involved in the classical glutathione transferase activity [20]. However, some changes could be detected in PcGSTFuA3 (serine to glycine), CcGSTFuA6820 (serine to asparagine), and in the proteins belonging to the PcGSTFuA5 subclass (serine to alanine) suggesting differential catalytic properties (Figure 2).

In previous work a sulphate binding pocket has been identified in the crystal structure of PcGSTFuA1. The sulphate ion was located near the catalytic residue Ser-22 and was stabilized by the side chains of Tyr-46 and Arg-153. Globally among GSTFuAs investigated in this study, these residues are well conserved, except for the CcGSTFuA2461 tyrosine, which is substituted by a phenylalanine (Figure 2).

### Protein production

To investigate functionally this GST class, four isoforms of *P. chrysosporium* were produced in *E. coli* and purified. Unfortunately PcGSTFuA5 could not be produced whatever the culture conditions used (solid or liquid cultures, static or agitated conditions, rich (malt) or minimal medium, wood or anthracene as substrate). In addition, five isoforms from *C. cinereus* were selected over the 14 encoded in the genome, to represent the general diversity of GSTFuA found in this organism. Unfortunately, even if the homologue of PcGSTFuA5 (CcGSTFuA6672) was successfully amplified and expressed in *E.coli*, the resulting protein remained insoluble despite various tested protocols, preventing biochemical characterization of any isoform belonging to this subclass.

### Structural diversity

**Figure 3:**
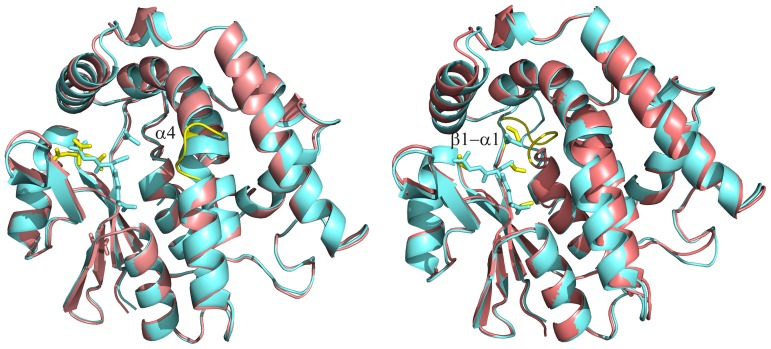
Views of the superpositions of PcGSTFuA1-2 monomers (left) and PcGSTFuA1-3 monomers (right). PcGSTFuA1 cartoon is colored aquamarine while PcGSTFuA2-3 cartoons are colored salmon. The ‘fold diversities’ in PcGSTFuA1-3 isoforms are highlighted in yellow : (left) the disruption of the helix α4 in PcGSTFuA2 ; (right) the loop β1-α1. Ligands are shown in stick mode. Glutathione and acetate molecules bound in PcGSTFuA1 are colored aquamarine. Citrate molecule (left) and formate molecules (right) molecules bound in PcGSTFuA2 and 3, respectively, are colored yellow.

Among the 5 existing isoforms in *P. chrysosporium*, two three-dimensional structures were resolved (PcGSTFuA2 and PcGSTFuA3). PcGSTFuA2 crystallized in the space group *C*222_1_ with three dimers in the asymmetric unit. Global NCS restraints were imposed during refinement because diffraction limit of the crystals was 3.2 Å. The structure of PcGSTFuA3 was solved at high resolution (2.1 Å) from crystals having the *P*6_1_22 space group with one polypeptide chain in the asymmetric unit. As assumed in our recent report [8] PcGSTFuA2, PcGSTFuA3 and PcGSTFuA1 share the same structure, which contains distinctive features when compared with canonical GST structure [4] (Figure 3) : (*i*) a new mode of dimerization, (*ii*) an original β-hairpin (β2’β2’’) that inhibits the formation of the regular GST dimer and acts as a lid over the G-site and (*iii*) a supplementary helix (α4’), between helices α4 and α5, that closes the presumed H-site.

**Figure 4:**
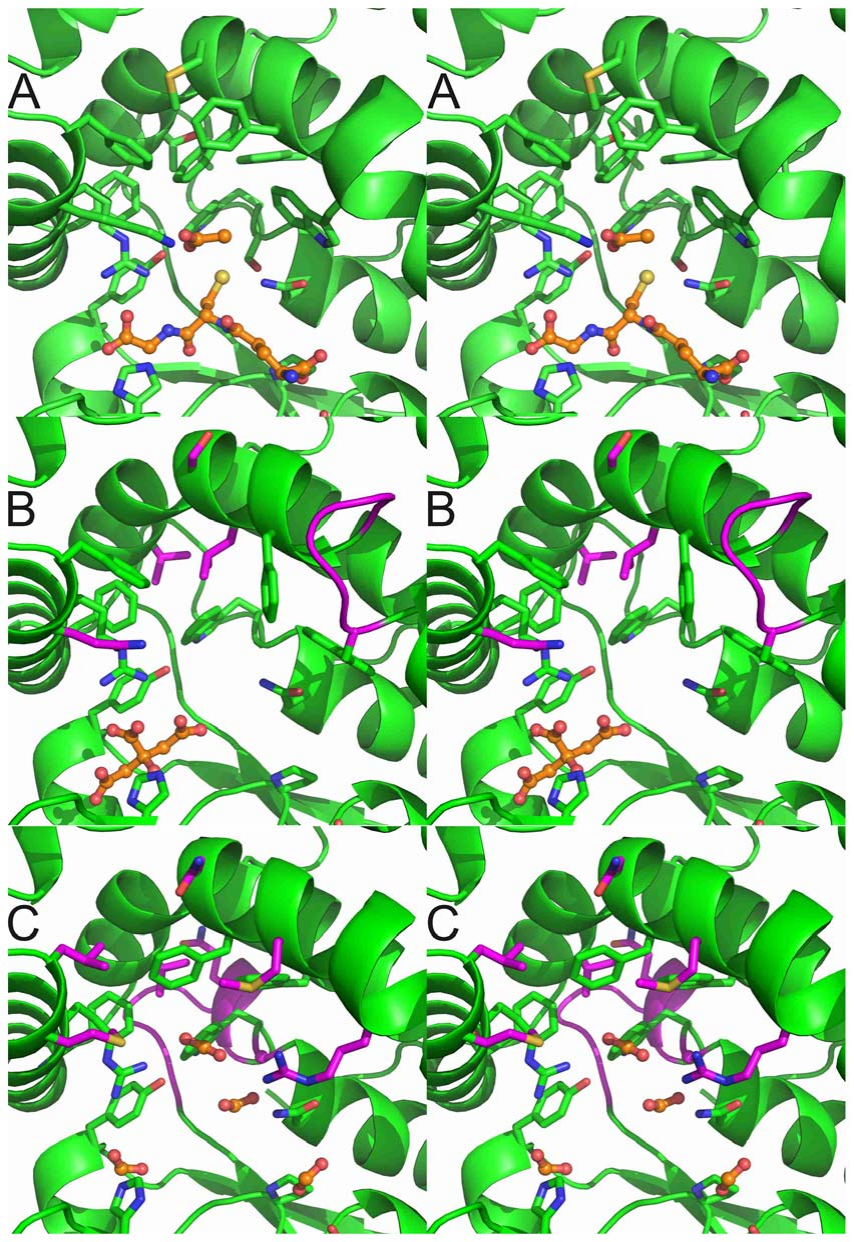
Stereo views of the active sites of PcGSTFuA1 (A), PcGSTFuA2 (B) and PcGSTFuA3 (C). The carbon atoms of the ligands are colored orange : (A) a glutathione molecule and an acetate ion ; (B) a citrate molecule ; (C) four formate molecules. Most of the carbon atoms are colored green. The carbon atoms, which are colored magenta, highlight structural diversities in PcGSTFuA2 (B) and in PcGSTFuA3 (C) in relation to PcGSTFuA1. Other atoms are colored according to their types.

In our recent study [8], we described the crystal structures of PcGSTFuA1 with and without GSH named apo and holo forms, respectively. The analysis of the models revealed significant conformational changes of the enzyme upon GSH binding. On the one hand the β-hairpin (β2’β2’’) closes the G-site and a number of residues modify their side-chain conformations to interact with glutathione. On the other hand two residues open slightly the H-site by changing their lateral-chain conformations. Structural superpositions of apo/holo PcGSTFuA1, PcGSTFuA2 and PcGSTFuA3 unambiguously showed that PcGSTFuA2 and PcGSTFuA3 are in the holo conformation in the conditions used. However, in both structures, no peak could be assigned to glutathione in the electron density maps. The observed holo conformations can be explained by the presence of crystallization agent molecules in the G sites, which mimic some of the interactions formed by bound GSH. A citrate ion and three formate molecules were assigned in the PcGSTFuA2 and PcGSTFuA3 structures, respectively (Figure 4).

The distance plot, which compares superposed structures of holo PcGSTFuA1 and PcGSTFuA2 (Figure S1), reveals no big differences. The largest is localized in the center of the helix α4 where a residue is inserted in PcGSTFuA2 sequence (Figures 3 and 4B). This difference results in a disruption in the continuity of helix α4. This region has to be considered as two helices in PcGSTFuA2 (α4A and α4B, Figure 3). It is worth noting that the disruption region is near the presumed H-site. This structural diversity could explain functional variabilities between PcGSTFuA1 and PcGSTFuA2. The distance plot, which compares holo PcGSTFuA1 and PcGSTFuA3, revealed a significant difference in the β1-α1 loop. It is linked to the low sequence identity between PcGSTFuA1 and PcGSTFuA3 in this region and to a one-residue shorter loop in PcGSTFuA3 (Figures 3 and 4C). The β1-α1 loop in PcGSTFuA3 is longer than that of almost all GSTs and reduces the solvent accessibility of the H-site as observed for β1-α1 loop in PcGSTFuA1 [8].

The comparison of the quaternary structures of PcGSTFuA1 and PcGSTFuA2 revealed little difference. The V-shaped PcGSTFuA3 dimer is roughly 5° smaller than that observed in PcGSTFuA1. Among the two hydrophilic patches involved in dimer stabilization, only one consists of residues conserved in both isoforms. The consequence is an increase in the number of intermolecular hydrogen bonds from 5 (in PcGSTFuA1 dimer) to 12 (in PcGSTFuA3) leading to a more stable dimer. The residues involved in the hydrophobic lock-and-key motif are well conserved in PcGSTFuA1, PcGSTFuA2 and PcGSTFuA3.

In the active site, Ser-22 (PcGSTFuA1 numbering), which catalyses the GSH addition on ITC in PcGSTFuA1, is present in PcGSTFuA2 but absent in PcGSTFuA3 (Figure 2). However, Asn-24 (PcGSTFuA1 numbering), which has been assumed to be involved in the peroxidase activity of PcGSTFuA1, is conserved in the three isoforms. This residue exhibits the same conformation in all three structures (Figure 4). Comparison of the active sites revealed that the residues involved in the GSH and in the sulphate binding sites in PcGSTFuA1 are well conserved and could play the same role in PcGSTFuA2 and PcGSTFuA3. In the structure of PcGSTFuA2, a citrate molecule mimics the binding of GSH glycyl moiety. The citrate ion stabilization involves the main chains of Tyr-71 and Val-73 and the side-chains of Tyr-46, His-70 and Arg-154 (Figure 4B). In PcGSTFuA3, three formate ions mimic the binding of the two GSH carboxylate groups and of the GSH thiolate group. A fourth formate molecule occupies the position of the sulphate ion in PcGSTFuA1 (Figure 4C). The structural variability is rather localized in the presumed electrophilic substrate binding site even though most of the residues are hydrophobic in the three isoforms. The local structure diversity is highlighted in Figure 4.

### Diversity of GSTFuAs activities

The catalytic pattern of the different enzymes was studied using various substrates. Glutathione transferase activity was tested using 1-chloro-2,4-dinitrobenzene (CDNB), phenethyl isothiocyanate (phenethyl-ITC) and 4-nitrophenyl butyrate (PNP-butyrate). PcGSTFuAs, although clustering together in our phylogenetic analysis, exhibited various catalytic patterns (Table 3). PcGSTFuA1 displayed various activities in contrast to PcGSTFuA2 (both proteins having 74% of identity), which remained inactive against all tested substrates. Moreover, PcGSTFuA1 and PcGSTFuA3 were active as GSH transferase with phenethyl-isothiocyanate and PNP-butyrate.

**Table 3:** Kinetic parameters of PcGSTFuAs and CcGSTFuAs in enzymatic assays.

Km (μM)	PcGSTFuA1	PcGSTFuA2	PcGSTFuA3	PcGSTFuA4	CcGSTFuA2461	CcGSTFuA6800	CcGSTFuA6801	CcGSTFuA6820
CDNB	ND	ND	ND	ND	4205±557	ND	1172±84.96	ND
Tertbutyl	ND	ND	ND	ND	ND	ND	ND	ND
Cumen	ND	ND	2037±556	ND	ND	ND	ND	ND
H2O2	ND	ND	ND	ND	ND	ND	ND	ND
HED	ND	ND	ND	ND	ND	ND	ND	ND
DHA	ND	ND	ND	ND	ND	ND	ND	ND
ITC	119.0±14.4	ND	53.16±5.37	ND	191.2±17.8	2431±254	41.56±3.73	ND
PNP-butyrate	774.3±88.3	ND	612.6±81.1	ND	ND	ND	177.7±8.9	ND
CMFDA (-GSH)	3.6±0.6	ND	ND	ND	ND	0.153±0.023	0.945±0.074	ND
CMFDA (+GSH)	8.0±0.8	1.07±0.16	5.1±0.5	1.442±0.07	ND	5.05±1.29	3.13±0.73	ND
GSH	216.4±34.9	ND	21.1±3.6	ND	435.0±29.4	474.9±43.8	167.3±10.5	ND
								
kcat (min^−1^)	PcGSTFuA1	PcGSTFuA2	PcGSTFuA3	PcGSTFuA4	CcGSTFuA2461	CcGSTFuA6800	CcGSTFuA6801	CcGSTFuA6820
CDNB	ND	ND	ND	ND	443.2±39.6	ND	251.9±8.1	ND
Tertbutyl	ND	ND	ND	ND	ND	ND	ND	ND
Cumen	ND	ND	76.70±9.78	ND	ND	ND	ND	ND
H2O2	ND	ND	ND	ND	ND	ND	ND	ND
HED	ND	ND	ND	ND	ND	ND	ND	ND
DHA	ND	ND	ND	ND	ND	ND	ND	ND
ITC	510.9±16.0	ND	130.9±2.9	ND	368.1±10.3	469.2±29.9	1065.0±20.2	ND
PNP-butyrate	165.0±5.6	ND	246.5±10.2	ND	ND	ND	115.7±1.4	ND
CMFDA (-GSH)	0.006±0.0002	ND	ND	ND	ND	0.123±0.003	0.343±0.005	ND
CMFDA (+GSH)	0.17±0.04	0.033±0.001	0.65±0.02	0.002±0.0001	ND	0.39±0.05	0.85±0.08	ND
GSH	662.0±14.2	ND	52.33±1.68	ND	46.01±2.45	37.15±1.6	31.95±0.53	ND
								
kcat/Km (min^−1^/μM^−1^)	PcGSTFuA1	PcGSTFuA2	PcGSTFuA3	PcGSTFuA4	CcGSTFuA2461	CcGSTFuA6800	CcGSTFuA6801	CcGSTFuA6820
CDNB	ND	ND	ND	ND	0.11±0.07	ND	0.21±0.09	ND
Tertbutyl	ND	ND	ND	ND	ND	ND	ND	ND
Cumen	ND	ND	0.037±0.017	ND	ND	ND	ND	ND
H2O2	ND	ND	ND	ND	ND	ND	ND	ND
HED	ND	ND	ND	ND	ND	ND	ND	ND
DHA	ND	ND	ND	ND	ND	ND	ND	ND
ITC	4.3±0.3	ND	2.46±0.54	ND	1.92±0.58	0.19±0.11	25.62±5.41	ND
PNP-butyrate	0.21±0.01	ND	0.40±0.12	ND	ND	ND	0.65±0.16	ND
CMFDA (-GSH)	0.0017±0.0002	ND	ND	ND	ND	0.82±0.13	0.36±0.06	ND
CMFDA (+GSH)	0.021±0.002	0.031±0.006	0.13±0.04	1.39*10^−3^±0.14*10^−3^	ND	0.077±0.038	0.27±0.11	ND
GSH	3.1±0.4	ND	2.48±0.47	ND	0.11±0.08	0.078±0.036	0.19±0.05	ND

The apparent K_m_ values for all compounds were determined using a concentration range of 0.1–10 mM in the presence of 5 mM GSH. The K_m_ value for GSH was determined with 1 mM Phenethyl-ITC for PcGSTFuA1, PcGSTFuA3, CcGSTFuA2461, CcGSTFuA6801 and 50 µM CMFDA for CcGSTFuA6800 with a concentration range of 0.01 to 10 mM GSH. The apparent K_m_ and k_cat_ values were calculated by nonlinear regression using the Michaelis-Menten equation (r^2^>0.99). Data are represented as mean ± S.D. (n±3). ND: Not Detected. The detection limit was estimated at 0.5 mUI.

This catalytic variability was also observed when comparing the *C. cinereus* isoforms. CcGSTFuA2461, CcGSTFuA6800 and CcGSTFuA6801 were active as GSH transferase against phenethyl-ITC, but only CcGSTFuA2461 and CcGSTFuA6801 were active with the universal GST substrate CDNB. In addition, CcGSTFuA6801 was able to cleave the ester linkage of PNP-butyrate.

None of the tested GSTFuAs exhibited thiol transferase activity using HED and DHA as substrates. PcGSTFuA3 was the only tested protein active against cumene hydroperoxide, whereas CcGSTFuA6820 remained inactive whatever the tested substrate.

CMFDA is a molecule releasing fluorescence upon activation by esterases [21]. As shown earlier [8], PcGSTFuA1 displayed activity against CMFDA, in presence or in absence of GSH. This feature is not shared by the other tested PcGSTFuAs as they exhibited an esterase activity only in presence of GSH (Table 3). Activity of CcGSTFuAs against CMFDA has been also tested demonstrating that only CcGSTFuA6800 and CcGSTFuA6801 were able to cleave this substrate either in absence or presence of GSH.

### Fluorescent probes to screen the ligandin property of GSTFuAs

**Figure 5:**
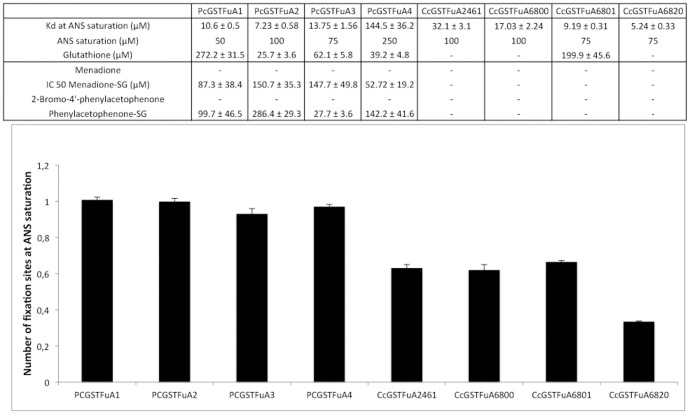
ANS binding and competition experiments onto PcGSTFuAs and CcGSTFuAs. (A) K_d_ values were determined by plotting concentration of ANS bound onto GSTFuAs against concentration of free ANS. Values given for glutathione, menadione-SG and phenylacetophenone-SG the values given are IC50 obtained by fitting data to equation 2 described in Material and Methods. (B) Number of binding sites for PcGSTFuAs and CcGSTFuA determined by fitting data to Equation 1.

8-anilino-1-naphtalene sulfonic acid (ANS) is an environment sensitive fluorescent dye used to characterize hydrophobic sites of proteins [22]. This probe has been successfully used to analyze the ligandin properties of PcGSTFuA1 [8]. Using a similar approach, we have tested ANS binding onto the different recombinant proteins, following the appearance of a characteristic fluorescence emission signal at 475 nm (excitation at 375 nm). Globally, all GSTFuAs were able to bind ANS, but some properties, i.e. number and position of the binding sites, varied on the different isoform (Figure 5). The number of ANS binding sites, determined by fitting data of saturation experiments to Equation 1 detailed in material and methods section, is approximately equal to 1 for PcGSTFuAs, whereas CcGSTFuAs exhibited values below 0.7 suggesting a lower affinity of these *C. cinereus* isoforms for the probe.

The ANS binding site onto PcGSTFuA1 has been shown to overlap the glutathione binding site [8]. Similarly, GSH inhibits ANS binding onto PcGSTFuAs and CcGSTFuA6801. The IC50 values determined after fitting the obtained data to Equation 2 (see materials and methods) are reported in Figure 5. These measured values are in accordance with the Km of GSH found in the enzymatic assays for PcGSTFUA1/3 and CcGSTFUA6801. In addition, PAP-SG and menadione-SG were able to inhibit ANS binding only for PcGSTFuAs, while the same compounds deprived of the GSH adduct could not. These results demonstrate that ANS binding overlaps at least partially the glutathione binding site onto PcGSTFuAs. In CcGSTFuAs this peculiar feature is not conserved anymore since, except for CcGSTFuA6801, GSH or glutathionylated compounds did not inhibit ANS binding.

**Figure 6:**
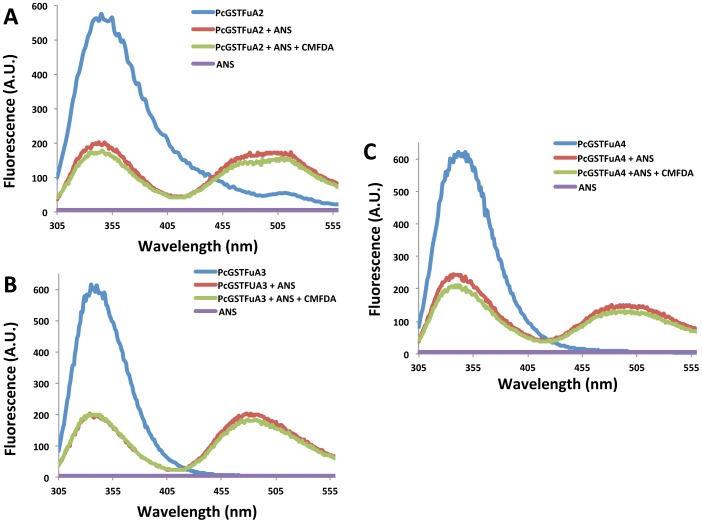
Tryptophan based fluorescence experiments illustrating ANS binding site upon addition of substrate onto (A) PcGSTFuA2, (B) PcGSTFuA3, (C) PcGSTFuA4. Upon tryptophan excitation at 290 nm, FRET between 100/75/250 µM ANS and 3 µM PcGSTFuA2/3/4 is characterized by the apparition of a signal at 475 nm (red) and is not altered in presence of 50 µM CMFDA (green). Emission spectra of ANS alone and PcGSTFuAs alone are colored in purple and blue respectively.

To determine the ANS binding site onto PcGSTFuAs, we used the fluorescence properties of tryptophan (Figure 6). Tryptophan fluorescence of PcGSTFuAs was measured using 290 nm and 340 nm as excitation and emission wavelengths respectively in presence or absence of ANS. Fluorescence resonance energy transfer (FRET), revealed by the appearance of a signal at 475 nm, was observed between Trp and ANS confirming the binding of ANS onto the proteins, the energy transfer being highly dependent on the distance between the donor and acceptor molecules. Taking advantage of the FRET phenomena in presence of ANS, competition experiments have been performed to investigate putative overlap between the H-site and ANS binding site. From kinetic data, all PcGSTFuAs exhibit a high affinity for CMFDA with GSH (Table 3). ANS binding onto PcGSTFuAs remained unaltered in presence of CMFDA demonstrating that the ANS binding site does not interfere with CFMDA binding site (Figure 6). Taken together these results suggest that specific ANS binding at the G-site, as described previously for PcGSTFuA1, is a well-conserved feature in this class for *P.chrysosporium*. In contrast, for CcGSTFuAs, this feature is not so well conserved as shown by the lack of competition for protein binding between GSH or glutathionylated compounds and ANS in these isoforms, except for CcGTSFuA6801.

In order to investigate potential ligandin properties of GSTFuAs (i.e. determination of L-site), we used 4-Methylumbelliferyl acetate (MUA) as a probe, which requires the cleavage of one ester bond before its detection by fluorescence. Interestingly, all GSTFuAs were able to cleave MUA even in absence of glutathione, however with a very small catalytic efficiency (Table 4). Interestingly, the only enzyme inactive in the tested enzymatic assays (CcGSTFUA6820) turned out to be active against this compound without GSH, demonstrating that it still possesses the ability to recognize hydrophobic compounds. Addition of glutathione with MUA led to two distinct behaviours: (i) a loss or a strong alteration of the ability to cleave the ester bond of MUA (CcGSTFuA2461, CcGSTFuA6820, CcGSTFuA6800, PcGSTFuA2, PcGSTFuA3 and PcGSTFuA4) (Table 4) (ii) a strong increase in catalytic efficiency upon GSH addition without alteration of the apparent affinity towards MUA (CcGSTFuA6801, PcGSTFuA1).

**Table 4:** Kinetic parameters of PcGSTFuAs and CcGSTFuAs towards MUA in absence or presence of GSH.

Km (μM)	PcGSTFUA1	PcGSTFUA2	PcGSTFUA3	PcGSTFUA4
MUA (−GSH)	262.8±33.7	23.5±6.2	33.89±4.78	51.56±7.09
MUA (+GSH)	206.7±23.3	216.1±45.2	372.1±53.9	N.D
kcat (min^−1^)	PcGSTFUA1	PcGSTFUA2	PcGSTFUA3	PcGSTFUA4
MUA (−GSH)	0.095±0.004	0.302±0.006	0.182±0.003	0.208±0.004
MUA (+GSH)	57.83±2.90	1.27±0.14	29.65±2.36	N.D
kcat/Km (min^−1^/um^−1^)	PcGSTFUA1	PcGSTFUA2	PcGSTFUA3	PcGSTFUA4
MUA (−GSH)	0.36*10^−3^±0.12*10^−3^	0.0128±0.0001	5.37*10^−3^±0.62*10^−3^	4.03*10^−3^±0.56*10^−3^
MUA (+GSH)	0.28±0.12	5.87 10^−3^±3.1 10^−3^	0.079±0.044	N.D
Km (μM)	CcGSTFuA2461	CcGSTFuA6800	CcGSTFuA6801	CcGTSFuA6820
MUA (−GSH)	38.7±7.1	25.39±4.37	182.3±29.4	16.9±2.1
MUA (+GSH)	N.D	111.7±11.1	189.7±7.3	N.D
kcat (min^−1^)	CcGSTFuA2461	CcGSTFuA6800	CcGSTFuA6801	CcGTSFuA6820
MUA (−GSH)	0.026±0.001	0.065±0.002	0.070±0.004	0.078±0.002
MUA (+GSH)	N.D	5.10±0.18	7.79±0.13	N.D
kca/Km (min^−1^/um^−1^)	CcGSTFuA2461	CcGSTFuA6800	CcGSTFuA6801	CcGTSFuA6820
MUA (−GSH)	0.67*10^−3^±0.14*10^−3^	2.56*10^−3^±0.46*10^−3^	0.38*10^−3^±0.13*10^−3^	4.61*10^−3^±0.9*10^−3^
MUA (+GSH)	N.D	0.046±0.016	0.041±0.017	N.D

The apparent K_m_ values were determined using a concentration range of 100–800 µM in absence (−GSH) or presence of 5 mM GSH (+ GSH). The apparent K_m_ and k_cat_ values were calculated by nonlinear regression using the Michaelis-Menten equation (r^2^>0.99). Data are represented as mean ± S.D. (n±3). ND: Not Detected. The detection limit was estimated at 0.5 mUI.

These results demonstrate that except in CcGSTFuA6801 and PcGSTFuA1, MUA and glutathione binding sites overlap at least partially onto the tested proteins. In conclusion, experiments using either ANS or MUA demonstrate that the overlap of G-site and L-site is a conserved feature among the tested GSTFuAs.

### Diversity of GSTFuAs Ligandin properties

Using ANS or MUA as screening tools, competition experiments have been performed for PcGSTFuAs and CcGSTFuAs with 11 structurally variable compounds. Similarly to what was observed in enzymatic tests, GSTFuAs exhibited various ligandin profiles (Table 5). CcGSTFuA6820 was able to bind 8 compounds; PcGSTFuA1 and CcGSTFuA6801 were able to bind 7 compounds; PcGSTFuA2/3 and CcGSTFuA2461 were able to bind 4 while PcGSTFuA4 was able to bind 3 of them. Among these products, those recognized by all PcGSTFuAs are mostly small aldehydes while CcGSTFuAs mainly recognized larger hydrophobic compounds or phenolic acids. Steric hindrance is a factor influencing ligand binding since epicatechin and catechin hydrate, two of the largest compounds used, are both bound by PcGSTFuA1, CcGSTFuA6801 and CcGSTFuA6820 but not by other GSTFuAs.

**Table 5:** Competition experiments between ANS or MUA and wood compounds.

	PcGSTFuA1	PcGSTFuA2	PcGSTFuA3	PcGSTFuA4
Fluorescent probe	ANS	ANS	ANS	ANS
Coniferaldehyde	62.3±1.2 µM	115.6±44.1 µM	68.4±25.3 µM	146.6±5.1 µM
Vanillin	217.8±8.5 µM	84.7±5.2 µM	134.0±7.6 µM	308.9±29.3 µM
Methoxybenzophenone	-	-	-	-
4-chloro-3-nitrobenzoic acid	1.5±0.1 mM	1.3±0.2 mM	234.7±56.7 µM	-
4'-hydroxyacetophenone	2.2±0.4 mM	-	-	-
Gallic acid	-	-	-	-
Vanillic acid	-	-	-	-
Epicatechin	1.6±0.1 mM	-	-	-
Syringaldehyde	62.5±1.6 µM	89.3±2.8 µM	37.2±3.8 µM	198.5±25.2 µM
Catechin hydrate	575.3±98.7 µM	-	-	-
Metoxycinnamic	-	-	-	-
				
	CcGSTFuA2461	CcGSTFuA6800	CcGSTFu6801	CcGSTFuA6820
Fluorescent probe	MUA	ANS/MUA	ANS	MUA
Coniferaldehyde	-	ND	327.4±50.6 µM	768.8±98.4 µM (non-competitive)
Vanillin	-	ND	423.6±107.3 µM	-
Methoxybenzophenone	-	ND	-	-
4-chloro-3-nitrobenzoic acid	287.4±29.7 µM (competitive)	ND	2.3±1.3 mM	1.3±0.03 mM (non-competitive)
4'-hydroxyacetophenone	352.1±25.1 µM (competitive)	ND	2.9±1 mM	1.2±0.03 mM (non-competitive)
Gallic acid	113.9±8.9 µM (competitive)	ND	-	566.4±16.5 µM (non-competitive)
Vanillic acid	1±0.1 mM (competitive)	ND	-	-
Epicatechin	-	ND	3.9±2.3 mM	1.4±0.04 mM (non-competitive)
Syringaldehyde	-	ND	275.8±97.8 µM	594.2±37.6 µM (non-competitive)
Catechin hydrate	-	ND	3.7±1.7 mM	564.2±12 µM (non-compet)
etoxycinnamic	-	ND	-	1.5±0.04 mM (non-competitive)

For ANS competition, the values given are IC50 obtained by fitting data to equation 2 described in Material and Methods.

For MUA competition, the values given are K_i_ obtained by fitting data to noncompetitive or competitive inhibition of esterase activity in the presence of 0 to 10 mM inhibitor.

The apparent K_i_ values were calculated by nonlinear regression using the competitive or noncompetitive inhibitions equations described in Material and Methods (r^2^>0.99). Data are represented as mean ± S.D. (n±3). ND: Not determined since no competition was observed with GSH for ANS and MUA.

Taken together, our results suggest that the ligandin function linked to the G-site is present in enzymes of the GSTFuA class which can bind a variety of ligands especially in *C. cinereus*.

## Discussion

Using comparative genomics, we show here that the GSTFuA class is widely distributed within Agaricomycotina. The expansion of this GST class seems to be linked to the fungal ability to cope with recalcitrant organic matter as wood or litter, independently of the degradation strategy (litter degraders, white or brown rotters). Among saprotrophic basidiomycetes, GSTFuA sequences cluster according to taxonomy suggesting a recent diversification of this fungal class as already observed for the fungal Ure2p class [23], [24]. Nevertheless, the ubiquity of GSTFuAs among saprotrophic basidiomycetes could suggest putative important physiological functions in these organisms.

Our biochemical and structural studies have been performed with eight isoforms produced from two different agaricomycetes: *P. chrysosporium* and *C. cinereus*. The obtained data demonstrate a large catalytic versatility of GSTFuAs associated with little structural diversity. GSTFuAs exhibited indeed different catalytic profiles, some of them being able to act as GSH transferases against various substrates (phenethyl-ITC, CDNB and PNP-butyrate), peroxidases and also esterases. However none presented thiol transferase activity. Surprisingly, three isoforms were inactive against the classical GST substrates, with nevertheless a weak esterase activity against CMFDA for two of them. These results suggest that evolution of multigenic families, and especially GSTs, results in a specialization of the various isoforms. Indeed, GSTs are often referred to as promiscuous enzymes with low specificity. Here our data suggest one clear primary function (conjugation of GSH with isothiocyanates) together with some other lower level activities (peroxidase or esterase), which can be regarded as a vestige of the broad specificity of ancestors or an emerging function due to neofunctionalization events.

Sequence alignment and structural data indicate that three potential residues could be involved in catalysis: Ser22, Asn24 and Tyr46 (PcGSTFuA1 numbering). Ser22 is involved in glutathionylation activity of GSTFuA1 [8], Asn24 seems to be crucial for the peroxidase activity of *Saccharomyces cerevisiae* Ure2p [25], while Tyr46 stabilizes the sulphate ion in PcGSTFuA1 [8]. None of these residues are strictly conserved within the GSTFuAs investigated in this study, in agreement with the different catalytic profiles observed. The mutant PcGSTFuA1 S22A generated in a previous study revealed the complex relationship between this catalytic diversity and primary sequence variability. Indeed, the resulting enzyme, which gained thiol transferase and peroxidase activities exhibited a catalytic profile relatively similar to PcGSTFuA3 that possesses a glycinyl residue at the same position.

The inability of some of these enzymes to act efficiently as transferases raises the question of their function in the fungal cell. One particular feature of PcGSTFuA1 is the presence of a ligand binding site overlapping the G-site. This putative L-site is responsible for the binding of small aromatic compounds [8]. Here we show by using fluorescent probes that this feature is conserved in the eight studied isoforms. As for enzymatic activities, GSTFuAs exhibited versatility in this ligandin property. Variations of this ligandin property were highlighted by competition experiments between ANS or MUA and small aromatic compounds. In these competition assays, all GSTFuAs investigated possessed their own ligandin profile, including those that were inactive in enzymatic assays. Previously identified L-sites in GSTs are located in the dimer interface for the mu-class of the parasitic worm *Schistosoma japonicum* [26], the squid sigma-class [27], and the human omega class [6], while it overlaps the H-site for the phi-class of *Arabidopsis thaliana* [28] or the human mu-class [29]. Our competition experiments with fluorescent probes demonstrated that this L-site, in the GSTFuA class, is located in the vicinity of the G-site without overlapping the H-site, and is able to recognize a large variety of compounds possessing different chemical functions.

Saprotrophic fungi such as *P. chrysosporium* or *C. Cinereus* are constantly exposed to small aromatic compounds such as phenolic acids and phenolic aldehydes derived from lignin. Since these fragments are further metabolized and mineralized intracellularly, fungi should possess versatile intracellular metabolic systems. *C. Cinereus* is capable of degrading phenolic lignin model compounds such as syringic acid or vanillic acid [30], whereas in the white-rot basidiomycete *P. chrysosporium* several genes encoding intracellular are induced by exogenous addition of lignin related compounds such as vanillin [31] or benzoic acid [32]. Addition of benzoic acid induced genes involved in aromatic compound metabolism/xenobiotic detoxification, such as cytochromes monooxygenases, while addition of vanillin is responsible for the activation of fungal metabolic pathways of phenolic compounds [33]. However, vanillin is not a nutrient substrate but rather acts as a chemical stress on fungal cells [31]. Altogether, these observations demonstrate the existence of a xenobiotic stress-derived metabolic response system towards compounds resulting from lignin degradation. This balance between slight chemical stress and catabolism implies that fungi must develop a way to deal with the myriad of small aromatic compounds resulting from ligninolysis. For this purpose, GSTFuAs have apparently evolved from needs related more to ligand recognition than conjugation of these compounds. Indeed, some isoforms were unable to conjugate GSH onto the tested hydrophobic compounds but possessed a ligandin property at least for these tested compounds. This property could thus be helpful to protect the cell against reactivity of lignin degradation compounds depending on the intracellular concentration of GSH and wood compounds. The variability observed among GSTFuAs might have been driven by survival strategies to provide a superior metabolic system to cope with lignin and its derivatives as it has already been noticed for cytochromes monooxygenases (P450) of *P. chrysosporium* [34].

Another example of driven evolution concerns the P450s from plants and insects. Plants have evolved P450s implicated in biosynthesis pathways of secondary metabolites as defence against insects while insects have evolved P450s to counter the toxic effects of these plants metabolites. Both evolutionary pathways involve continual gene duplications and mutations resulting in alteration of substrate range and catalytic properties [35].

In conclusion, we postulate that the versatility and promiscuity of the extended GSTFuA class in saprotrophic fungi reflects the adaptation of these organisms to their lifestyle.

## Supporting Information

Figure S1 Distance between equivalent Cα positions after superpositions of apo/holo PcGSTFuA1, PcGSTFuA2 (top) and of apo/holo PcGSTFuA1 and PcGSTFuA3 (bottom). Pair-wise RMS deviation of corresponding Cα atoms after superposition of apo PcGSTFuA1 and PcGSTFuA2 is 0.84 Å and after superposition of holo PcGSTFuA1 and PcGSTFuA2 is 0.61 Å. Pair-wise RMS deviation of corresponding Cα atoms after superposition of apo PcGSTFuA1 and PcGSTFuA3 is 1.04 Å and after superposition of holo PcGSTFuA1 and PcGSTFuA3 is 0.88 Å.
(TIFF)

Table S1 Distribution of GSTFuAs. Sequences were obtained from the Joint Genome Institute with Blastp using all *P. chrysosporium* PcGSTFuA sequences as template in all available genomes of chytridiomycotina, mucoromycotina, saccharomycotina, pezizomycotina, puccinomycotina, ustilagomycotina and agaricomycotina.
(DOCX)
