# Supplementary figures and images for: Diversification of Fungal Specific Class A Glutathione Transferases in Saprotrophic Fungi
Source: PLoS One. 2013 Nov 20;8(11):e80298. doi: 10.1371/journal.pone.0080298 (PMC3835915; doi:10.1371/journal.pone.0080298)

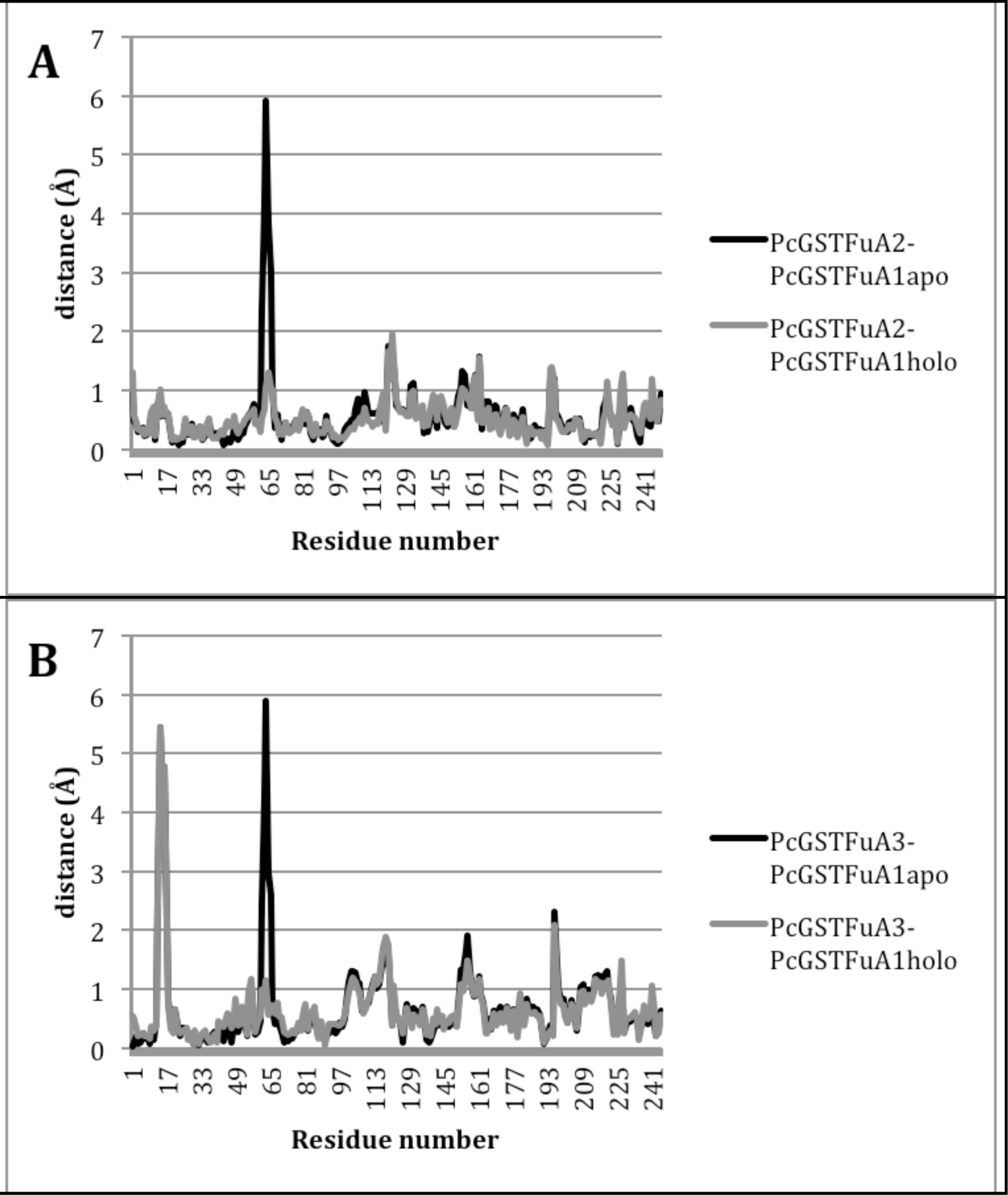

Supplement: Figure S1 — Distance between equivalent Cα positions after superpositions of apo/holo PcGSTFuA1, PcGSTFuA2 (top) and of apo/holo PcGSTFuA1 and PcGSTFuA3 (bottom). Pair-wise RMS deviation of corresponding Cα atoms after superposition of apo PcGSTFuA1 and PcGSTFuA2 is 0.84 Å and after superposition of holo PcGSTFuA1 and PcGSTFuA2 is 0.61 Å. Pair-wise RMS deviation of corresponding Cα atoms after superposition of apo PcGSTFuA1 and PcGSTFuA3 is 1.04 Å and after superposition of holo PcGSTFuA1 and PcGSTFuA3 is 0.88 Å. (TIFF) [file pone.0080298.s001.tiff]
