# Supplementary material for: Diversification of Fungal Specific Class A Glutathione Transferases in Saprotrophic Fungi
Source: PLoS One. 2013 Nov 20;8(11):e80298. doi: 10.1371/journal.pone.0080298 (PMC3835915; doi:10.1371/journal.pone.0080298)
Supplement: Table S1 — Distribution of GSTFuAs. Sequences were obtained from the Joint Genome Institute with Blastp using all P. chrysosporium PcGSTFuA sequences as template in all available genomes of chytridiomycotina, mucoromycotina, saccharomycotina, pezizomycotina, puccinomycotina, ustilagomycotina and agaricomycotina. (DOCX) [file pone.0080298.s002.docx]

**Supplemental Table S1. Distribution of GSTFuAs.**

Sequences were obtained from the Joint Genome Institute with Blastp using all *P. chrysosporium* PcGSTFuA sequences as template in all available genomes of chytridiomycotina, mucoromycotina, saccharomycotina, pezizomycotina, puccinomycotina, ustilagomycotina and agaricomycotina.
